# KLF4 deletion alters gastric cell lineage and induces MUC2 expression

**DOI:** 10.1038/cddis.2016.158

**Published:** 2016-06-09

**Authors:** T Yu, X Chen, T Lin, J Liu, M Li, W Zhang, X Xu, W Zhao, M Liu, D L Napier, C Wang, B M Evers, C Liu

**Affiliations:** 1 Markey Cancer Center, University of Kentucky, Lexington, KY 40506, USA; 2 Nanfang Hospital, Southern Medical University, Guangzhou 510515, China; 3 Department of Surgery, University of Kentucky, Lexington, KY 40506, USA; 4 Department of Molecular and Cellular Biochemistry, University of Kentucky, Lexington, KY 40506, USA

## Abstract

Gastric cancer is one of the most common types of cancer in the world, particularly in underdeveloped countries. The mechanism of gastric cancer is less understood compared with other types of gastrointestinal (GI) cancers. Krüppel-like factor 4 (KLF4) is a zinc-finger transcription factor and is a potential tumor suppressor in GI cancers. In this study, we have generated two mouse models, *Rosa-Cre;Klf4*^*fl/fl*^ and *Lgr5-Cre;Klf4*^*fl/fl*^. KLF4 was deleted by Rosa-Cre in the gastric epithelia cells or by Lgr5-Cre in the antral stem cells in the adult mice. KLF4 deletion resulted in increased proliferating cells and decreased pit mucous cells. Surprisingly, the intestinal goblet cell marker, MUC2, which is not expressed in normal gastric tissues, was strongly induced at the base of the KLF4-deleted antral glands. To understand the clinical relevance of these findings, we analyzed the expression of KLF4 and MUC2 in human gastric cancer. In a subset of human gastric cancer, the expression of KLF4 is negatively associated with MUC2 expression. In conclusion, KLF4 is essential for normal homeostasis of antral stem cells; loss of KLF4 and expression of MUC2 could be important markers for gastric cancer diagnosis.

Gastric cancer is currently the fourth most common cancer in the world. Globally, gastric cancer accounts for 989 000 new cases and over 700 000 cancer deaths each year.^1^ The 5-year survival from gastric cancer is as low as 20–30%,^2^ due to oftentimes non-specific symptoms that often delay the diagnosis. The common mechanisms of gastric cancer are still obscure. Understanding these mechanisms is essential for gastric cancer diagnosis and treatment.

The gastric epithelium is a continuous self-renewing tissue.^3^ The stem cells in the gastric gland are required for epithelial cell proliferation and differentiation.^4, 5^ Recently, Lgr5^+ve^ cells were identified as stem cells for both intestine and stomach.^6, 7^ In the adult stomach, *Lgr5* is expressed mainly in the antrum and rarely in the corpus or fundus. Lgr5^+ve^ stem cells are restricted to the base of antral glands. These stem cells drive self-renewal in the stomach and build long-lived gastric units *in vitro.*^6^ Deletion of the tumor suppressor gene *APC* in Lgr5^+ve^ cells activates Wnt signaling and induces tumor formation in the distal stomach,^6^ consistent with earlier studies of aberrant Wnt signaling in gastric cancer.^8, 9^

In addition to Lgr5^+ve^ stem cells, a rare population of ‘label-retaining' cells with multilineage potential were identified in the antrum.^10^ These quiescent gastric progenitor cells (villin^+ve^) express villin and were located at or below the isthmus region of the antral gland.^10^ Recently, Krüppel-like factor 4 (KLF4) was deleted in the villin^+ve^ cells by villin-Cre. KLF4 deletion enhanced chemical-induced gastric carcinogenesis.^11^ KLF4 is a zinc-finger protein highly expressed in the skin and gut.^12^ As a transcription factor, KLF4 has multiple functions. For example, KLF4 has an essential role in regulating embryonic stem cells and inducing pluripotent stem cells (iPS cells).^13^ The function of KLF4 in the intestine has been well studied. In the stomach, KLF4 has been deleted by Foxa3-Cre during the embryonic stage. At 6–12 months, altered proliferation and differentiation were observed in the gastric body.^14^ However, the function of KLF4 in the Lgr5^+ve^ cell lineage in the antrum has not been investigated. Given that 60–80% of intestinal-type gastric carcinomas initiate in the antrum,^15, 16^ it is important to determine KLF4 function in this region, especially in the stem cells, which may contribute to both gastric cancer and metaplasia.

In this study, we established two new mouse models. In the first model, KLF4 was deleted in the mice using Rosa-Cre. In a second model, KLF4 was deleted in the Lgr5^+ve^ stem cell in the adult mice using Lgr5-Cre. These models allowed us to analyze KLF4 function in the proliferation and differentiation of adult stem cells, without affecting early development.^17^ We found that KLF4 had a key role in maintaining antral stem cell homeostasis. Importantly, we found abundant MUC2-positive cells at the base of antral glands but not in the corpus after KLF4 deletion. The expression of KLF4 and MUC2 was further analyzed in human gastric cancer tissues and adjacent normal tissues. KLF4 was downregulated in gastric cancer, probably by epigenetic regulation. MUC2 was not detected in normal tissues but overexpressed in a subset of gastric cancer, indicating that KLF4 and MUC2 could be potential markers for gastric cancer diagnosis.

## Results

### Rosa-Cre-mediated KLF4 deletion induced proliferation of antrum and corpus of adult mice

**Figure 1:**
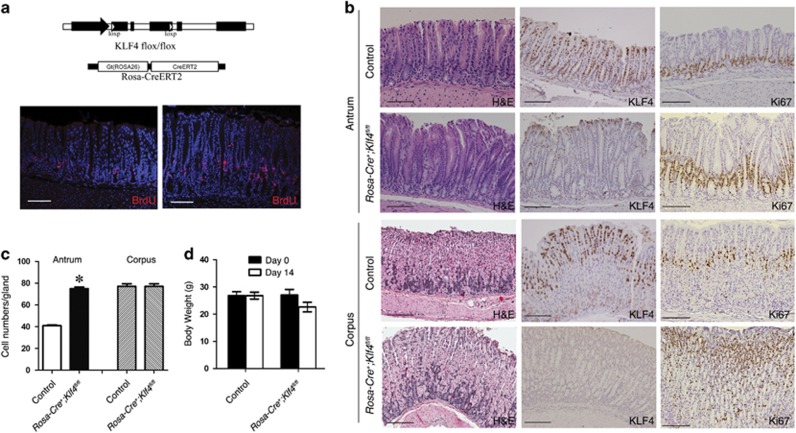
Rosa-Cre-mediated KLF4 deletion in the gastric antrum and corpus of adult mice. (**a**) Top: Schematic diagram of the genome of *Rosa-Cre;Klf4* ^*fl/fl*^ mice. Bottom: BrdU labeling of mouse antrum from the control and the KLF4-deleted mice. Scale bar: 100 *μ*m. (**b**) H&E, Ki67 and KLF4 staining in the antrum and corpus of control *Klf4* ^*fl/fl*^ mice and *Rosa-Cre*^*+*^*;Klf4* ^*fl/fl*^ mice. KLF4 was expressed in the pit cells and upper glands in the control mice and was deleted in most antral cells in the *Rosa-Cre*^*+*^*;Klf4* ^*fl/fl*^ mice. Scale bar: 100 *μ*m. (**c**) Statistic comparison of cell numbers in the antral glands and corpus glands of control mice and the KLF4-deleted mice. Data are represented as mean±S.D. (*P<0.001). (**d**) Body weight of control and KLF4-deleted mice at day 0 and day 14 of tamoxifen induction. Data are represented as mean±S.D. Four control mice and four *Rosa-Cre*^*+*^*;Klf4* ^*fl/fl*^ mice were tested in this experiment

To study the function of KLF4 in the stomach, we established the *Rosa-Cre*^*+*^*;Klf4*^*fl/fl*^ mouse model by crossing the *Klf4*^*fl/fl*^ strain with *Rosa-Cre*^*+*^ strain (Figure 1a, top). ROSA-Cre, which is expressed in both the antrum and the corpus, can be activated by tamoxifen. Two weeks after tamoxifen treatment, KLF4 was efficiently deleted both in the antrum and in the corpus as indicated by KLF4 immunohistochemical analyses (Figure 1b). H&E staining demonstrated that KLF4 deletion significantly changed the morphology of the corpus and antral glands (Figure 1b). Ki67-positive cells were increased in both antrum and corpus, and expanded from bottom towards the mid-region of gastric glands, suggesting that KLF4 deletion enhanced gastric cell proliferation. BrdU labeling experiment also indicated an increase in cell proliferation in the antrum of KLF4-deleted mouse (Figure 1a, bottom). The KLF4-deleted antral glands were more elongated than the control glands. The average cell numbers in the KLF4-deleted glands were significantly increased in the antrum but not in the corpus (Figure 1c). By the time of 2-week induction, body weights of the mice were slightly while not significantly decreased (Figure 1d). In addition, as observations for longer effect of KLF4 depletion, at 2-month induction, we observed ulcerative dermatitis lesions in both shoulders and on the lateral sides of the mice. The affected skin has gotten very tight, which limited their ability to groom themselves and to close their bottom jaws. These pathological observations could be due to loss of function of KLF4 in the skin,^12, 17^ in addition to the defects in the GI. Thus, we focused on the effects of KLF4 deletion on stomach after 2-week tamoxifen treatment.

### Rosa-Cre-mediated KLF4 deletion changed gastric cell lineage of adult mice

**Figure 2:**
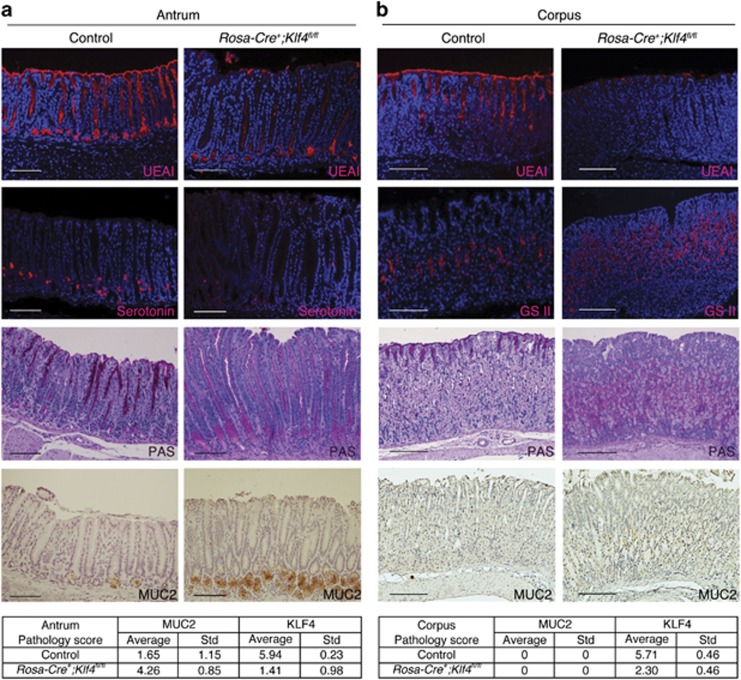
Rosa-Cre-mediated KLF4 deletion changed gastric cell lineage of adult mice (**a**) and corpus (**b**) region of adult mice. UEA I was expressed in the pit mucous cells in the control antrum and corpus and was significantly decreased in the antrum and corpus of KLF4-deleted mice. Expression of gastric endocrine cell marker, serotonin, is decreased in the antrum of *Rosa-Cre*^*+*^*;Klf4* ^*fl/fl*^ mice. GSII-positive cells were significantly increased in the middle region of KLF4-deleted corpus. PAS staining was decreased in the pit mucous cells and increased in the middle to lower part of KLF4-deleted cells. MUC2 expression is induced in the antrum of the *Rosa-Cre*^*+*^*;Klf4* ^*fl/fl*^ mice. Scale bar: 100 *μ*m. Three control mice and three *Rosa-Cre*^*+*^*;Klf4* ^*fl/fl*^ mice were tested in this experiment. Average pathology score sums up the intensity score and the percentage score from IHC staining. ‘Std', standard deviation

In addition to changes in cell proliferation and morphology, we analyzed the changes in cell lineage by immunofluorescent staining. Cell differentiation marker Ulex Europeus Agglutinin I (UEA I) expression was decreased in the KLF4-deleted antrum and corpus (Figures 2a and b), suggesting that KLF4 regulates the differentiation of pit mucous cells in the antrum and the corpus. The number of serotonin-positive cells, a marker for gastric enteroendocrine cells, was decreased in the KLF4-deleted antral glands (Figure 2a), suggesting that KLF4 regulates the differentiation of enteroendocrine cells in the antrum. Grifforia simplifolica II (GSII) lectin is a marker for neck mucous cells. GSII-positive cells were increased in the middle region of KLF4-deleted corpus glands (Figure 2b), indicating that KLF4 regulates the differentiation of neck mucous cells in the corpus. PAS staining indicates a shift of mucous cells both in the antrum and in the corpus, which was decreased in the pit mucous cells and increased in the middle region of the KLF4-deleted antrum and corpus (Figures 2a and b).

As PAS staining is a general marker for mucin-secreting cells, we did IHC staining for MUC2, which is a specific marker for intestinal goblet cells. MUC2 is not expressed in normal stomach but is expressed in both complete and incomplete intestinal metaplasia.^18^ Staining results showed a significant increase in MUC2-positive cells at the base of antral glands, suggesting that KLF4 deletion leads to differential expression of mucin genes, or different mucous cell lineages (Figure 2a). MUC2 expression was not detected in the corpus (Figure 2b). Pathology scores of MUC2 and KLF4 demonstrated a strong correlation between KLF4 deletion and MUC2 induction in the antrum at 2 weeks post KLF4 deletion (Figure 2, bottom). Similar results were observed at 2 months upon KLF4 deletion (not shown).

### Potential mechanisms of KLF4 downregulation in human gastric cancer

**Figure 3:**
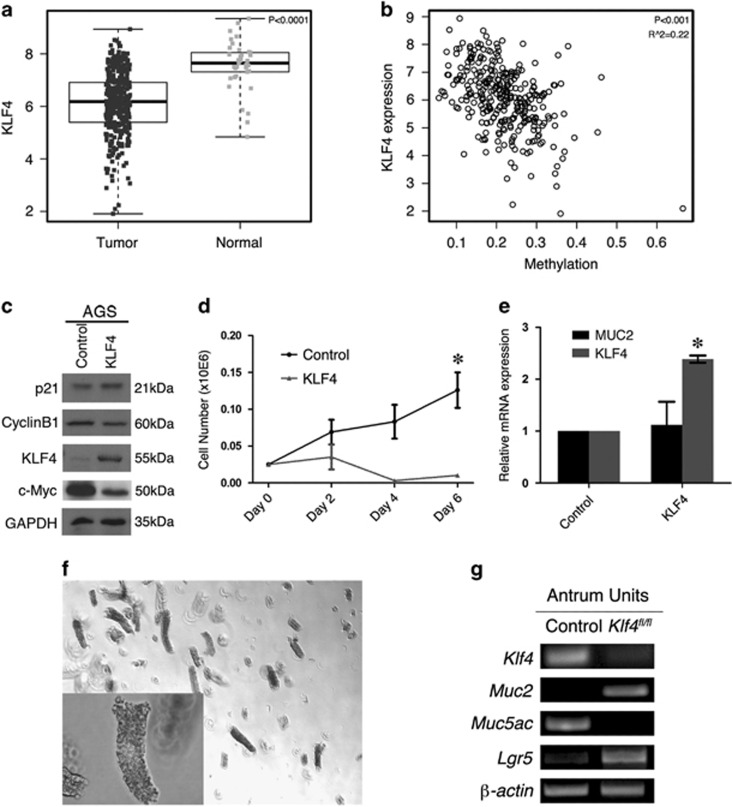
KLF4 is a potential tumor suppressor in human gastric cancer. (**a**) Statistical analysis of KLF4 expression in normal and gastric cancer from TCGA database. (**b**) Statistical analysis of correlation between expression level and methylation of KLF4 in gastric cancer patients. (**c**) Western blot of KLF4 and target genes in AGS cells infected with control or KLF4-carrying adenovirus. (**d**) Growth curves of AGS cells infected with control or KLF4-carrying adenovirus. Data are represented as mean±S.D. (*P<0.005). (**e**) Real-time PCR of MUC2 and KLF4 mRNA expression in AGS cells infected with control or KLF4-carrying adenovirus. Data are represented as mean±S.D. (**f**) Representative image of antral glands isolated from adult mice. (**g**) RT-PCR analysis of gene expression in *Klf4*-deleted antral glands. Three control mice and three *Rosa-Cre*^*+*^*;Klf4* ^*fl/fl*^ mice were used in this experiment

As KLF4 deletion enhanced gastric cell proliferation, KLF4 may acts as a tumor suppressor in gastric cancer. We analyzed gastric cancer data from The Cancer Genome Atlas (TCGA) database and found that KLF4 was significantly downregulated in tumor samples (Figure 3a). To investigate the mechanisms of KLF4 downregulation in gastric cancer, we analyzed the promoter methylation of KLF4 in TCGA database and found a negative correlation between methylation and expression level of KLF4 (Figure 3b), suggesting that DNA methylation is a potential mechanism for the silence of KLF4 in human gastric cancer. To test the function of KLF4 in human gastric cancer cells, we infected AGS cell line with adenovirus carrying KLF4, which induces overexpression of KLF4 in the cells. We analyzed the expression of several KLF4 target genes. Exogenous expression of KLF4 only affected the expression of c-Myc (Figure 3c). AGS cell proliferation was significantly inhibited by KLF4 expression (Figure 3d), further supporting that KLF4 is a tumor suppressor in gastric cancer. We analyzed MUC2 expression in AGS cells using real-time PCR, as mucins are extremely large, which makes them difficult to test with western blot. However, we did not see any change in MUC2 expression (Figure 3e), suggesting that KLF4 does not directly control MUC2 transcription in gastric cancer cells. In the animal model, KLF4 deletion may alter the differentiation of mucus cells, thus inducing MUC2 expression at the base of antral glands (Figure 2a). To further test the effect of KLF4 deletion on target gene expression, antral glands were isolated from the control mice and *Rosa-Cre*^*+*^*;Klf4*^*fl/fl*^ mice 2 weeks after tamoxifen injection (Figure 3f). As expected, KLF4 was lost after Cre activation (Figure 3g). MUC2 expression was induced, while MUC5AC expression was decreased in the *Klf4*-deleted glands (Figure 3g). These results are consistent with the PAS staining and MUC2 IHC results, suggesting that *Klf4* deletion leads to differential expression of mucin genes, or different mucous cell lineages. Additionally, we found that *Klf4* deletion upregulated Lgr5 expression, thus supporting our previous finding that KLF4 represses Wnt signaling, which controls Lgr5 expression.^19, 20, 21^

### Lgr5-Cre-mediated KLF4 deletion induced MUC2 in the antrum of adult mice

**Figure 4:**
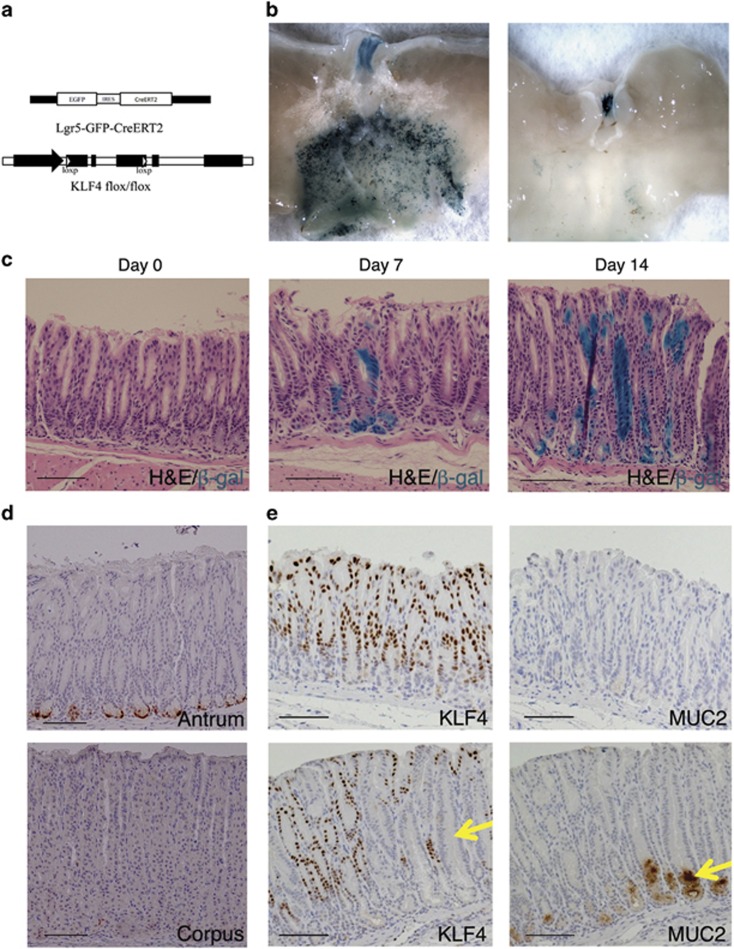
KLF4 deletion in Lgr5^+ve^ cells in the gastric antrum of adult mice. (**a**) Schematic diagram of the genome of *Lgr5-Cre;Klf4* ^*fl/fl*^ mice. (**b**) Whole-mount images of stomach stained for LacZ. The LacZ staining was restricted to the antrum region in *Lgr5-Cre*^*+*^*;Rosa-LacZ* mice, and was negative in the *Lgr5-Cre*^-^*;Rosa-LacZ* mice. (**c**) Lineage tracing in *Lgr5-Cre*^*+*^*;Klf4* ^*fl/fl*^*;Rosa-LacZ* mice. LacZ-positive progeny of the Lgr5^+ve^ cells populates the antral glands 1 week and 2 weeks after tamoxifen induction. (**d**) Lgr5-GFP-ires-CreER fusion protein was expressed at the base of the antral glands in adult mice. (**e**) KLF4 and MUC2 expression in the antrum of control *Klf4* ^*fl/fl*^ mice (top) and *Lgr5-Cre*^*+*^*;Klf4* ^*fl/fl*^ mice (bottom). MUC2 expression was induced in the KLF4-deleted glands (arrows). Scale bar: 100 *μ*m

Gastric stem cells control proliferation and differentiation of gastric glands. We hypothesize that KLF4 may regulate Lgr5^+ve^ gastric stem cells. To specifically delete KLF4 and to study its function in these stem cells, we utilized the *Lgr5* knock-in mouse model.^7^ Lgr5-EGFP-ires-CreERT2 fusion protein is controlled by Lgr5 promoter (Figure 4a). To delete KLF4, the *Lgr5-Cre* mouse strain was crossed with the *Klf4*^*fl/fl*^ mouse strain (Figure 4a). To test the activity of Cre recombinase, the *Lgr5-Cre* mouse strain was crossed with the ROSA-lacZ reporter strain.^22^ Lgr5-Cre mice were treated with tamoxifen in order to activate the ROSA-LacZ reporter. LacZ expression was detected throughout the antral region, but not the corpus or fundic regions of *Lgr5-Cre*^*+*^*;Rosa-LacZ* mice (Figure 4b). In the lineage tracing studies, LacZ-positive cells ascended from the base to the mid-top regions of the antral glands in 1–2 weeks (Figure 4c), indicating that the recombination occurred in the Lgr5^+ve^ stem cells, which drive self-renewal of the antral glands. As detected by anti-GFP Ab, the Lgr5 fusion protein was expressed in cells located at the base of antral glands (Figure 4d), indicating the location of Lgr5^+ve^ stem cells.^6^ Consistent with previous studies,^6^ no GFP staining was noted in the corpus (Figure 4d). IHC staining indicated that KLF4-positive cells were located in most of the antral glands, especially in the upper 2/3 of the glands, while in *Lgr5-Cre*^*+*^*;Klf4*^*fl/fl*^ mice, KLF4 expression was absent in a number of glands 1–2 weeks after tamoxifen treatment (Figure 4e, left). In consistent with the *Rosa-Cre*^*+*^*;Klf4*^*fl/fl*^ mouse model, MUC2 was significantly increased at the base of KLF4-deleted antral glands of the *Lgr5-Cre*^*+*^*;Klf4*^*fl/fl*^ mice (Figure 4e, right). These results demonstrate that lack of KLF4 promotes an intestinal metaplasia-like phenotype, as judged by the staining for MUC2. Further investigation is of great interest to determine whether this is the result of misregulation of the Lgr5^+ve^ population.

### MUC2 is overexpressed in a subset of gastric cancer

**Figure 5:**
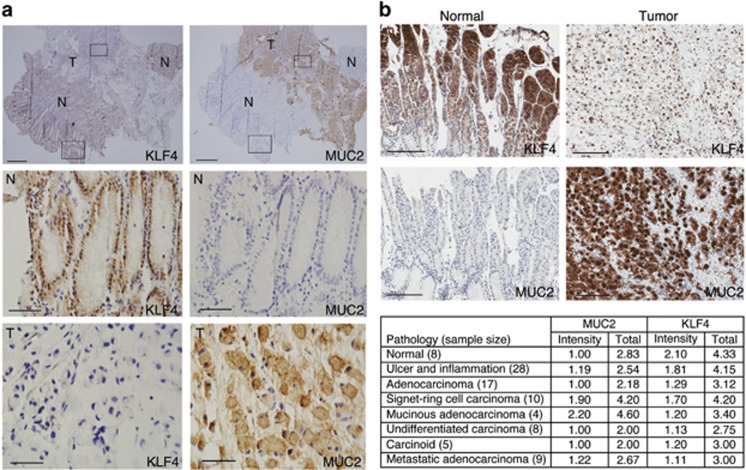
MUC2 is induced in human gastric cancer. (**a**) Representative images of a human signet ring cell carcinoma, showing decreased KLF4 expression (left) and increased MUC2 expression (right) in tumor sections. Scale bars for top panels: 200 *μ*m. Scale bars for middle and bottom panels: 50 *μ*m. N, normal tissue; T, tumor tissue. (**b**) Top: IHC staining of human gastric cancer tissue and normal gastric tissue with KLF4 and MUC2 antibodies. Scale bars: 100 *μ*m. Bottom: Average scores of KLF4 and MUC2 expression in human gastric cancer TMA

As KLF4 deletion induced MUC2 expression in the mouse antrum (Figures 2a and 4e) and KLF4 levels are decreased in human gastric cancer (Figure 3a), we analyzed MUC2 expression in a preliminary study using 11 gastric cancer samples provided by NanFang Hospital. We found that MUC2 staining was positive in 4 of 11 samples, including 3 signet ring carcinomas and 1 well-differentiated adenocarcinoma. KLF4 expression is negatively correlated with MUC2 in these signet ring carcinomas (Figure 5a, ‘T': tumor tissue). Signet ring cell carcinoma is a special subtype of mucinous adenocarcinoma, which accounts for about 20% of gastric cancer. In the adjacent normal mucosa, KLF4 is strongly expressed while the MUC2 staining is negative (Figure 5a, ‘N': normal tissue). However, the correlation between KLF4 and MUC2 is hard to define due to lack of sample size.

To further examine the correlation between KLF4 and MUC2 in human gastric cancer, and to understand the clinical relevance of these two proteins, we purchased TMA slides composed of 81 tumor samples and 8 normal tissues from the gastric cancer patients. To determine the correlation, we analyzed KLF4 and MUC2 expression by IHC staining. In normal tissue, KLF4 is intensely expressed while the expression of MUC2 could not be detected (Figure 5b). We statistically analyzed the TMA scores by grading the expression levels of KLF4 and MUC2, respectively (Figure 5b,Supplementary Figure S1). We found that signet ring cell carcinoma and mucinous adenocarcinoma have the highest total score of MUC2, while KLF4 score is variable in these tumors due to different localization of KLF4 in the tissues (Figure 5b).

**Figure 6:**
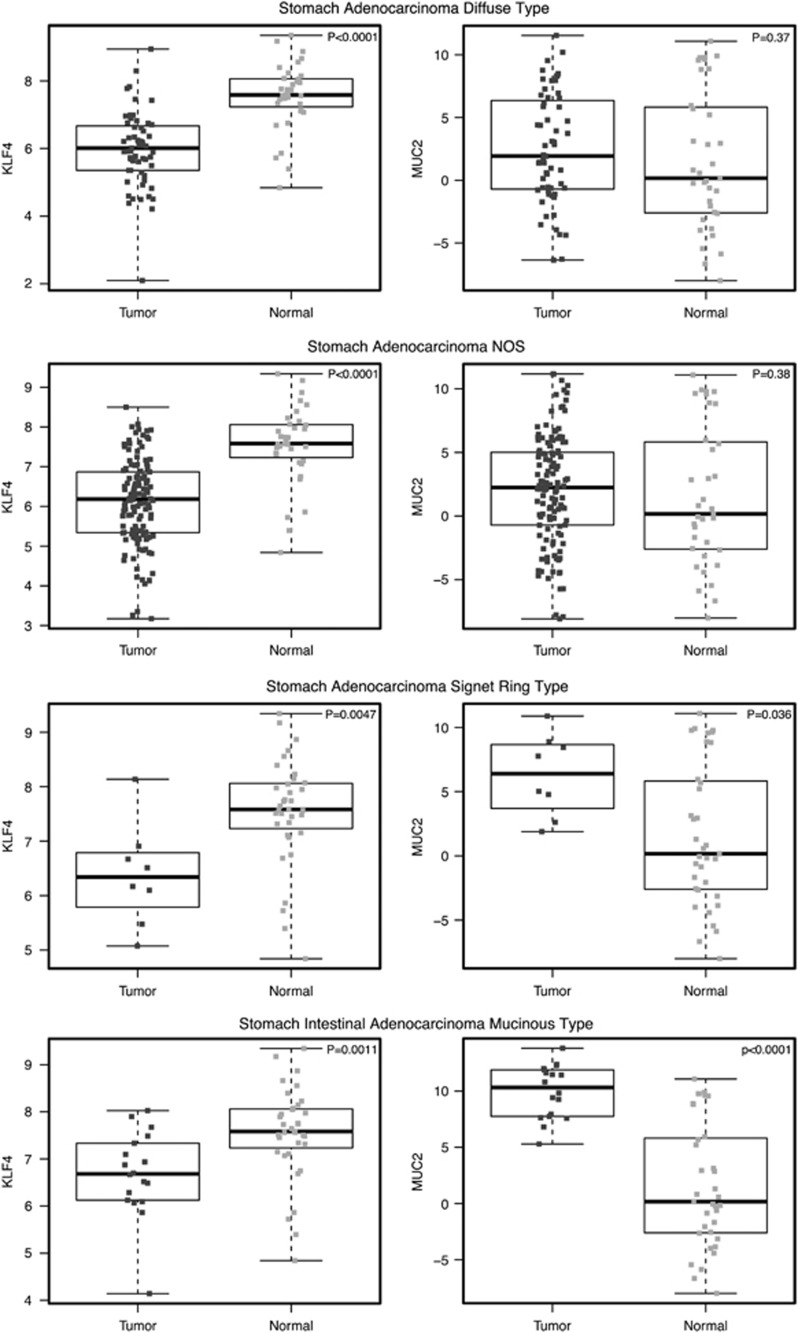
Statistical analysis of KLF4 and MUC2 expression in different types of gastric cancer. MUC2 expression is inversely correlated with KLF4 expression in a subset of human gastric cancer. NOS, not otherwise specified

To better define their correlation, we analyzed the expression levels of KLF4 and MUC2 from TCGA stomach adenocarcinoma database. In consistent with the TMA results, KLF4 was downregulated in all subtypes of stomach adenocarcinoma, while MUC2 was found to be induced in mucinous type of stomach intestinal adenocarcinoma (Figure 6), further indicating the effect of KLF4 deletion in inducing MUC2 expression and in facilitating formation of this particular type of adenocarcinoma.

## Discussion

KLF4 is a key regulator of cell proliferation and differentiation and has a tumor suppressor role in many cancers. KLF4 function in the stomach has been studied using foxa3-Cre and villin-Cre mouse models.^11, 14^ In these models, KLF4 was deleted in gastric cells at early developmental stages and the phenotypes were analyzed at adult stages. These are excellent models to study KLF4 function in the gastric development and tumorigenesis. To study how KLF4 regulates the cell lineage of adult stem cells, specifically, the Lgr5^+ve^ cells, we generated two mouse models using inducible Cre recombinase. Upon tamoxifen treatment, KLF4 can be rapidly deleted in gastric epithelial cells by Rosa-Cre, or specifically deleted by the Lgr5-Cre in the Lgr5^+ve^ stem cells and their daughter cells. The Lgr5-Cre model is more specific but KLF4 is only deleted in a limited number of antral glands. Thus, the Rosa-Cre and Lgr5-Cre models are complementary models to study KLK4 function in the adult stomach.

Under normal physiologic conditions, the adult gastric epithelium undergoes self-renewal with a balance of proliferation and apoptosis, which is regulated by multiple signal transduction pathways, such as Wnt, hedgehog and Notch.^6, 23, 24^ Wnt signaling regulates the self-renewal of Lgr5^+ve^ stem cells, which can generate a complete antral gland *in vivo* and *in vitro*. Abnormal activation of the Lgr5^+ve^ stem cells induces gastric tumorigenesis.^6^ When KLF4 was deleted in the antral glands, cell proliferation was increased and cell lineage was significant changed (Figure 1), suggesting that KLF4 may regulate proliferation and differentiation of gastric stem cells. Interestingly, KLF4 deletion significantly induced MUC2 expression in the antrum of both Rosa-Cre and Lgr5-Cre mouse models. MUC2 is an important marker for gastric cancer and intestinal metaplasia. However, its expression was not detected in the previous mouse models,^11, 14^ suggesting that inducible Cre is a valuable tool to study the homeostasis of adult antral glands.

The antral glands contain abundant mucous cells and enteroendocrine cells;^3^ KLF4 deletion enhanced cell proliferation and reduced the numbers of pit mucous cells and enteroendocrine cells in the antrum (Figure 2). KLF4 deletion by Lgr5-Cre and Rosa-Cre induced precancerous changes but did not induce tumor formation within 2 weeks of KLF4 deletion. Using a similar model, it has been reported that *Apc* deletion by Lgr5-Cre rapidly induces tumor formation.^6^ In previous studies using the villin-Cre model, gastric tumors were detected at age 35–80 weeks,^11^ indicating that long-term KLF4 deletion could induce gastric tumors, probably by enhancing other oncogenic pathways.

Several studies demonstrated that MUC2 is expressed in intestinal metaplasia and tumors at earlier stages.^25, 26^ We analyzed the expression of KLF4 and MUC2 in human gastric cancer samples and found a negative correlation between KLF4 and MUC2 levels in signet ring cell carcinoma and mucinous adenocarcinoma. Signet ring cell carcinoma is a special subtype of mucinous adenocarcinoma, which denotes a very poor prognosis. In fact, more patients with advanced signet cell carcinoma were diagnosed with lymph-node and ovarian metastases compared with patients with other types of gastric cancer. It is possible that KLF4 and MUC2 could be used as diagnostic markers in signet ring cell carcinoma.

In summary, loss of KLF4 in the gastric stem cells enhanced proliferation and disruption of homeostasis of gastric cell lineage. KLF4 deletion induced MUC2 expression in mouse stomach. These findings have important clinical relevance as the levels of KLF4 and MUC2 may be negatively correlated in a specific subset of human gastric cancer, and could be used as early diagnostic markers.

## Materials and Methods

### Animal studies

All animal studies were approved by Institutional Animal Care and Use Committee at the University of Kentucky. Mice of Lgr5-EGFP-ires-CreERT2 (*Lgr5-cre*) strain (Jackson Lab, Bar Harbor, ME, USA; 008875) were crossed with ROSA26-LacZ (*RosaL*) reporter strain (Jackson Lab, 003474) and *Klf4*^*flox/flox*^ (*Klf4*^*fl/fl*^) strain (MMRRC 029877-MU).^14^ Mice of Rosa26-CreERT2/L (*Rosa-cre*) strain (Jackson Lab, 008463) were crossed with *Klf4*^*fl/fl*^ strain. All mice used for experiments were on C57BL/6 genetic background. Mice were genotyped by PCR using cDNA samples from mice tails. For tamoxifen induction, mice were injected intraperitoneally with tamoxifen (Sigma, St. Louis, MO, USA) at 100 mg/kg body weight for 3 consecutive days, and killed 2 weeks post first injection. For BrdU labeling, 2 weeks after tamoxifen induction, mice were injected intraperitoneally once at 4-h intervals with 200 ml BrdU solution in PBS at 5 mg/ml before killing. Mouse stomach tissue was paraffin embedded and the micro-sections were double-stained with BrdU antibody.

### Cell culture and proliferation assay

Human gastric cancer cell line AGS was cultured in DMEM containing 10% fetal bovine serum and 1% penicillin/streptomycin. For proliferation assay, cells were plated at approximately 2.5 × 10^4^ cells per well in 12-well plates and counted at appropriate times using the cell viability analyzer (Beckman Coulter, Indianapolis, IN, USA; Vi-Cell XR). Cells were infected with vector-carrying or KLF4-carrying lentivirus, and equal numbers of cells were seeded onto 12-well plates 18 h post infection, thus counted as Day 0.

### Lectin staining

Specimens were deparaffinized and hydrated as previously described. Ulex europaeus agglutinin I (UEA I) (1 : 200; Vector Laboratories, Burlingame, CA, USA) or Griffonia simplicifolia II (GSII) (1 : 200; Vector Laboratories) lectin was incubated for 30 min at room temperature, followed by incubation with Texas Red Streptavidin (1 : 100; Vector Laboratories) for 10 min. Other procedures were the same as described for immunohistochemistry staining.

### Western blotting

Cells were lysed in the appropriate volume of lysis buffer (50 mM HEPES, 100 mM NaCl, 2 mM EDTA, 1% glycerol, 50 mM NaF, 1 mM Na_3_VO_4_, 1% Triton X-100, with protease inhibitors). The following antibodies were used: mouse anti-GAPDH (GeneTex, Irvine, CA, USA; GT239), rabbit anti-KLF4,^21^ and Bmi1 (Epitomics, Burlingame, CA, USA; S2983).

### Gastric units isolation and RT-PCR

Stomachs were isolated and opened along the greater curvature. After washing with cold PBS, the antrum was isolated under the microscope, divided into 5 mm pieces and was shaken slowly in 10 mM EDTA solution for 4 h at 4 °C. The fragments were transferred into 10 ml cold PBS and the glands were isolated by vigorous pipetting for 20 times, and centrifuged at 300 *g*/min for 5 min. The pellet was used for RNA extraction using the RNAeasy kit (Qiagen, Valencia, CA, USA), following the manufacturer's protocol, and reverse transcribed into cDNA using superscript reverse transcriptase.

### RT-PCR and real-time PCR

AGS cells were plated at approximately 2 × 10^5^ cells per well in a 6-well plate to be infected by vector-carrying or KLF4-carrying lentivirus. After 48 h of incubation, RNA was extracted using the RNeasy kit (Qiagen). Reverse transcriptase PCR (RT-PCR) was performed as described previously.^21^

Real-time RT-PCRs were carried out using SYBR Green PCR master mix reagents (Thermo, Waltham, MA, USA) on the ABI StepOnePlus Real-Time PCR System (Applied Biosystems, Life Technologies, Carlsbad, CA, USA). Thermal cycling was conducted at 95 °C for 10 min, followed by 40 cycles of amplification at 95 °C for 15 s and 60 °C for 1 min, then the melt curve at 95 °C for 15 s, 60 °C for 1 min and 95 °C for 15 s. The relative quantification of gene expression for each sample was analyzed by the △Ct method. The following primers were used: *β-actin*, 5′-CAACCGCGAGAAGATGAC-3′ and 5′-AGGAAGGCTGGAAGAGTG-3′ KLF4, 5′-AGAGGAGCCCAAGCCAAAG-3′ and 5′-CGTCCCAGTCACAGTGGTAAG-3′ MUC2, 5′-ACACCTGCTGCAACATTAC-3′ and 5′-CTGGCACTTGGAGGAATAAAC-3′ MUC5AC: 5′-CCATGAAGTGGGAGTGTG-3′ 5′-TTGGGATAGCATCCTTCCAG-3′. Lgr5: 5′-CCTACTTGACTTTGAGGAAGAC-3′ 5′-ATGTCCACTACCGCGATTAC-3′.

### Immunohistochemistry, immunofluorescent analysis and pathology scores

Sections (4 *μ*m) of formalin-fixed, paraffin-embedded specimens were deparaffinized in xylene and hydrated in graded alcohol. Antigen retrieval was performed with citric acid (pH 6.0) for 30 min in boiling water. Endogenous peroxidase was blocked by 3% hydrogen peroxide for 10 min at room temperature. The specimens were blocked by PBS (pH 7.4) containing 5% normal goat serum (Vector Laboratories) and then incubated in blocking solution (Vector Laboratories; SP2001), following the manufacturer's instructions. Samples were incubated with primary antibody overnight at 4 °C, and then incubated for 30 min at room temperature with peroxidase-conjugated anti-rabbit/anti-mouse/anti-rat IgG (1 : 500; Jackson Laboratories). After being incubated in diaminobenzidine (Vector Laboratories) for 30 s, sections were rinsed with distilled water and counterstained with Mayer's hematoxylin (Vector Laboratories). For immunofluorescent staining, after incubating with the primary antibodies, the proteins were detected by fluorescent secondary antibodies (1 : 100; Jackson Lab). Finally, sections were counterstained with DAPI (1 : 1000; Sigma). The primary antibodies used in this study were as follows: mouse anti-PCNA (1 : 100; Cell Signaling, Danvers, MA, USA); rat anti-Serotonin (1 : 100; Millipore, St. Charles, MO, USA); rabbit anti-MUC2 (1 : 400; Santa Cruz, Dallas, TX, USA). Rabbit anti-KLF4 (1 : 50) has been described previously.^21^ For pathology scores, the percentage of positive staining cells was scored as 0–3 (non=0, <10%=1, 10–50%=2, >50%=3), and intensity of the staining was scored as 0–3 (none=0, weak=1, intermediate=2, strong=3) as well. The total score sums up the intensity and the percentage score.

### Primers used for genotyping

The following primers were used: *Klf4*^*fl/fl*^: 5′-CTGGGCCCCCACATTAATGAG-3′, 5′-CGCTGACAGCCATGTCAGACT-3′ *Lgr5-Cre*: 5′-CTGCTCTCTGCTCCCAGTCT-3′, 5′-GAACTTCAGGGTCAGCTTGC-3′ *Lgr5* wild type: 5′-CTGCTCTCTGCTCCCAGTCT-3′, 5′-ATACCCCATCCCTTTTGAGC-3′ *Rosa-Cre*: 5′-AAAGTCGCTCTGAGTTGTTAT-3′, 5′-CCTGATCCTGGCAATTTCG-3′ and *Rosa* wild type: 5′-AAAGTCGCTCTGAGTTGTTAT-3′, 5′-GGAGCGGGAGAAATGGATATG-3′.

### Human tissue specimens

Human gastric adenocarcinoma samples were collected and analyzed in Nanfang Hospital in China. Both gastric adenocarcinomas and adjacent normal gastric mucosa (*n*=11 patients) were used for immunohistochemistry analyses. These studies were approved by the Research Ethics Committee of Nanfang Hospital, Southern Medical University and University of Kentucky.

### Statistical analysis

The statistical analysis for RNAseq and DNA methylation data was performed by R 3.2.3 (R Foundation for Statistical Computing, Vienna, Austria) and SAS 9.3 software (SAS Institute Inc., Cary, NC, USA). The normalized RNAseq gene expression data from The Cancer Genome Atlas (TCGA) Stomach adenocarcinoma (STAD) study were downloaded from the TCGA data portal (http://cancergenome.nih.gov) and log2-transformed. The data consisted of 373 tumor and 37 normal samples from a total of 377 patients. There were 33 patients that had both tumor and normal samples. The expressions of KLF4 and MUC2 in tumor and normal samples were compared based on a linear mixed model, which accounted for the correlation between tumor and normal samples from the same individual. The DNA methylation data (beta values) from the STAD study were also downloaded from the TCGA data portal. There were 309 patients that had both RNAseq and DNA methylation data. A linear regression model was used to assess the correlation between KLF4 mRNA expression and methylation. The statistical analysis for cell number counting in glands, body weights and cell proliferation assay was performed by GraphPad Prism 5 software (GraphPad Software, La Jolla, CA, USA). A Mann–Whitney non-parametric test was used for statistical comparison. Statistical significance was defined as *P*<0.05.

## Abbreviations

GI: gastrointestinal
KLF4: Krüppel-like factor 4
iPS cell: induced pluripotent stem cell
UEA I: Ulex Europeus Aglutinin I
GSII: Grifforia simplifolica II
TCGA: The Cancer Genome Atlas
IHC: immunohistochemistry
H&E: Hematoxylin-and-Eosin
