# Supplementary figures and images for: KLF4 deletion alters gastric cell lineage and induces MUC2 expression
Source: Cell Death Dis. 2016 Jun 9;7(6):e2255–. doi: 10.1038/cddis.2016.158 (PMC5143387; doi:10.1038/cddis.2016.158)

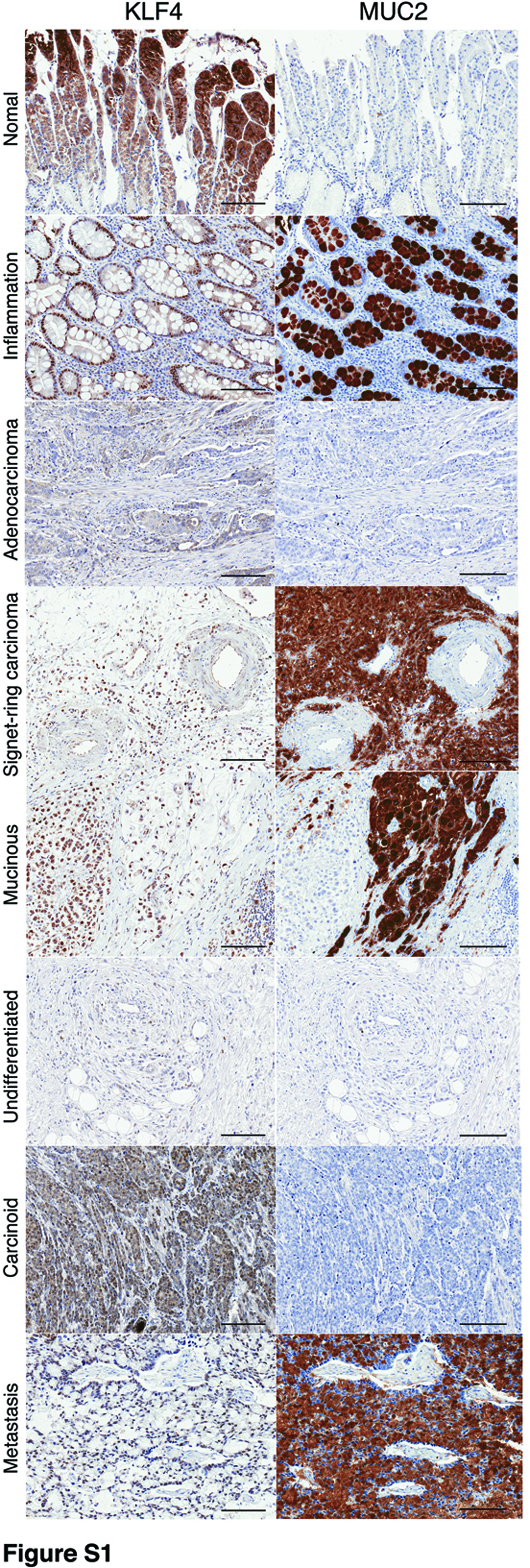

Supplement: Supplementary Figure S1 [file cddis2016158x1.tif]
