# Supplementary material for: KLF4 deletion alters gastric cell lineage and induces MUC2 expression
Source: Cell Death Dis. 2016 Jun 9;7(6):e2255–. doi: 10.1038/cddis.2016.158 (PMC5143387; doi:10.1038/cddis.2016.158)
Supplement: Supplementary Figure Legend [file cddis2016158x2.docx]

**Supplemental Figure Legend:**

**Supplemental Figure S1.** IHC staining of TMA slides of gastric cancer tissues and normal gastric tissues using KLF4 and MUC2 antibodies, respectively. Scale bar: 100μm.
